# Performance Evaluation of Cellulose Nanofiber with Residual Hemicellulose as a Nanofiller in Polypropylene-Based Nanocomposite

**DOI:** 10.3390/polym13071064

**Published:** 2021-03-28

**Authors:** Mohd Nor Faiz Norrrahim, Hidayah Ariffin, Tengku Arisyah Tengku Yasim-Anuar, Mohd Ali Hassan, Nor Azowa Ibrahim, Wan Md Zin Wan Yunus, Haruo Nishida

**Affiliations:** 1 Department of Bioprocess Technology, Faculty of Biotechnology and Biomolecular Sciences, Universiti Putra Malaysia, UPM, Serdang 43400, Malaysia; faiznorrrahim@gmail.com (M.N.F.N.); alihas@upm.edu.my (M.A.H.); 2 Research Center for Chemistry Defence, Universiti Pertahanan Nasional Malaysia, Kem Perdana Sungai Besi, Kuala Lumpur 57000, Malaysia; 3 Laboratory of Biopolymer and Derivatives, Institute of Tropical Forestry and Forest Products (INTROP), Universiti Putra Malaysia, UPM, Serdang 43400, Malaysia; tengkuarisyah@gmail.com; 4 Nextgreen Pulp & Paper Sdn. Bhd., Paloh Inai, Pekan 26600, Malaysia; 5 Department of Chemistry, Faculty of Science, Universiti Putra Malaysia, UPM, Serdang 43400, Malaysia; norazowa@upm.edu.my; 6 Centre for Defence Foundation Studies, Department of Chemistry and Biology, Universiti Pertahanan Nasional Malaysia, Kem Perdana Sungai Besi, Kuala Lumpur 57000, Malaysia; wanmdzin@upnm.edu.my; 7 Department of Biological Functions and Engineering, Graduate Kyushu Institute of Technology, School of Life Science and Systems Engineering, 2-4 Hibikino, Wakamatsu-ku, Kitakyushu, Fukuoka 808-0196, Japan; nishida@lsse.kyutech.ac.jp

**Keywords:** cellulose nanofiber, hemicellulose, nanocomposite, superheated steam, mechanical performance

## Abstract

Residual hemicellulose could enhance cellulose nanofiber (CNF) processing as it impedes the agglomeration of the nanocellulose fibrils and contributes to complete nanofibrillation within a shorter period of time. Its effect on CNF performance as a reinforcement material is unclear, and hence this study seeks to evaluate the performance of CNF in the presence of amorphous hemicellulose as a reinforcement material in a polypropylene (PP) nanocomposite. Two types of CNF were prepared: SHS-CNF, which contained about 11% hemicellulose, and KOH-CNF, with complete hemicellulose removal. Mechanical properties of the PP/SHS-CNF and PP/KOH-CNF showed an almost similar increment in tensile strength (31% and 32%) and flexural strength (28% and 29%) when 3 wt.% of CNF was incorporated in PP, indicating that hemicellulose in SHS-CNF did not affect the mechanical properties of the PP nanocomposite. The crystallinity of both PP/SHS-CNF and PP/KOH-CNF nanocomposites showed an almost similar value at 55–56%. A slight decrement in thermal stability was seen, whereby the decomposition temperature at 10% weight loss (*T*_d10%_) of PP/SHS-CNF was 6 °C lower at 381 °C compared to 387 °C for PP/KOH-CNF, which can be explained by the degradation of thermally unstable hemicellulose. The results from this study showed that the presence of some portion of hemicellulose in CNF did not affect the CNF properties, suggesting that complete hemicellulose removal may not be necessary for the preparation of CNF to be used as a reinforcement material in nanocomposites. This will lead to less harsh pretreatment for CNF preparation and, hence, a more sustainable nanocomposite can be produced.

## 1. Introduction

A nanocomposite is a material made from a combination of matrices such as polymer and nanofillers, aiming to create better properties compared to the individual material [1,2,3]. They generally consist of two phases, which are a matrix phase and a dispersed phase. Nanofillers such as fullerene [4], graphene [5], nanoclay [6], carbon nanotube [7], MXene [8] and cellulose nanofiber (CNF) are commonly used in nanocomposite production. The addition of these nanofillers in the polymer matrix has improved the thermal, flammability, flame retardancy and mechanical properties of composites. Among all these nanofillers, CNF has an advantage as compared to other nanofillers due to its renewability and biodegradability properties.

Recently, nanocomposites made from polymeric matrix and CNF have been the subject of interest due to their versatility to be used for many purposes, ranging from household goods, such as for packaging, to premium products, such as for bioadsorbents [9], packaging [10], the military [11], paper [12,13], biomedical applications [14,15,16], automotive applications [17], and electronics [18]. More specifically, CNF, which was derived from plants by intensive mechanical action, has gained interest as a superb reinforcement material owing to its superior properties such as high mechanical strength [19], high thermal stability [20], high crystallinity [21,22], and high surface area [23].

There have been numerous studies on the efficiency of CNF in increasing the mechanical strength of general-purpose thermoplastics such as polypropylene (PP) [24,25], polyethylene (PE) [22,26], polylactic acid (PLA) [27], epoxy/polysulfone [28], poly(ε-caprolactone) [29] and alcohol soluble phenolic [30]. Among many kinds of thermoplastics, PP is the most important and widely used polyolefin. Its low density, low production cost, design flexibility, and recyclability make it a popular choice as a matrix material for nanocomposites. In fact, numerous studies focusing on the effect of CNF towards PP aimed to improve its mechanical properties for various applications have been conducted [24,31,32].

Conventionally harsh treatments such as chemical, mechanical, thermal, or a combination thereof are needed to extract cellulose for CNF production. Chemical treatment is the most common method used to pretreat the lignocellulosic fiber. It requires the use of concentrated and high amounts of chemicals that are harmful to the environment. Moreover, this process is time and cost consuming. Scientists have continued to discover several greener alternatives to extract cellulose and, recently, focus is being given to lignocellulose-nanofiber (LCNF) to be used as a reinforcement material for nanocomposites.

The use of LCNF as a reinforcement material in PP has been reported by Iwamoto and colleagues [33]. LCNF is a CNF that does not solely consist of cellulose, but also other components such as hemicellulose, lignin, and silica. The presence of lignin is known to improve the mechanical performance and thermal stability of nanocomposites. Nevertheless, there is a lack of reports on the effect of residual hemicellulose in CNF on its performance as a reinforcement material in nanocomposites. 

The presence of hemicellulose in cellulose was found to assist in CNF nanofibrillation. Iwamoto and colleagues [34] reported that hemicellulose inhibits coalescence between cellulose fibrils during nanofibrillation, causing the CNF to be produced earlier at a higher yield. Nevertheless, the presence of hemicellulose in cellulose nanofiber may affect the properties of the CNF. For instance, it was reported that hemicellulose interrupted the nanocellulose fiber network and caused the nanocellulose film to have less tensile strength. This was observed for nanocellulose with high hemicellulose content at 27 wt.% [35]. This has led us to determine the effect of partial hemicellulose removal on CNF production, with the intention to assist in nanofibrillation and at the same time reduce the negative effect of hemicellulose. We previously reported that the presence of about 11% hemicellulose residue in the cellulose sample had assisted in nanofibrillation, whereby about 98% of the CNF produced had a width size of <100 nm after 10 cycles of wet disc milling. CNF with no hemicellulose had only 85% of nanofibrils with a width size of <100 nm [36]. The presence of hemicellulose avoided the aggregation of cellulose and, hence, more nanofibrils were produced. It is important to determine if this hemicellulose residue would affect the CNF properties as a reinforcement material in polymer nanocomposite samples. It is therefore important to evaluate the performance of the polymer nanocomposite reinforced with CNF with residual hemicellulose to clarify the effect of hemicellulose residue on the CNF properties. 

## 2. Materials and Methods

### 2.1. Materials

Oil palm mesocarp fiber (OPMF) was collected from Seri Hulu Langat Palm Oil Mill, Selangor, Malaysia. The fiber was first disintegrated, washed and dried. Sodium chlorite (NaClO_2_) and potassium hydroxide (KOH) were purchased from ACROS ORGANICS, Phillipsburg, NJ, USA and J.T Baker Neutracit, Phillipsburg, NJ, USArespectively. PP was purchased from Lyondellbasell (Saudi Polyolefins Company), Jubail, Saudi Arabia, while maleic anhydride-grafted-polypropylene (MA-g-PP) with an approximately 8–10 wt.% MA based on PP was purchased from Sigma Aldrich (M) Sdn. Bhd, Selangor, Malaysia. 

### 2.2. Fiber Pretreatment 

Cellulose isolation from OPMF was carried out as described in our previous study [36]. Lignin was removed by using NaClO_2_. Pretreatment for hemicellulose removal was conducted by two methods: superheated steam (SHS) pretreatment to partially remove the hemicellulose, and KOH pretreatment to completely remove the hemicellulose. The hemicellulose content of SHS-cellulose and KOH-cellulose was 11.4 and 0%, respectively, as reported in our previous report [12]. Both of the treated cellulose fibers were soaked in distilled water for 24 h prior to nanofibrillation to CNF. 

### 2.3. Preparation of Cellulose Nanofiber

Cellulose nanofiber was prepared by subjecting the SHS-cellulose and KOH-cellulose which had been soaked in water to nanofibrillation using a wet disk mill (WDM) (Multi mill, Grow Engineering, Adachi-ku, Tokyo, Japan) [36]. The cellulose suspension was passed through the WDM (Multi mill, Grow Engineering, Adachi-ku, Tokyo, Japan) equipped with two grinding stones at a rotational speed of 1800 rpm. The gap between the grinding stones was narrowed to 50 μm from the initial contact distance. After nanofibrillation, the CNF suspension was stored in a sealed container at 4 °C for further use in nanocomposite production.

### 2.4. Preparation of Nanocomposite

#### 2.4.1. Compounding

**Figure 1:**
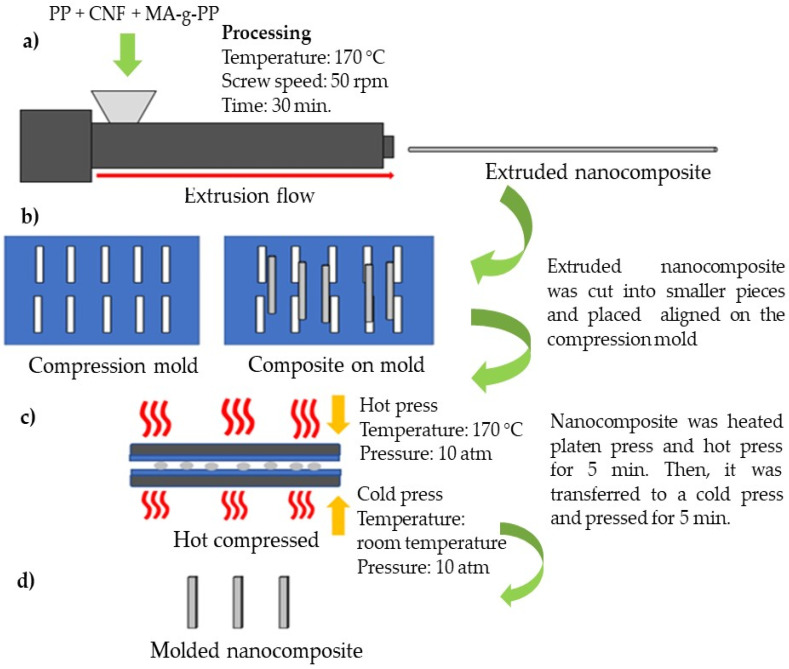
Processing flow of nanocomposite production by the twin-screw extruder.

Nanocomposites were compounded by using a twin-screw extruder (Imoto machinery Co., Ltd. Model IMO-160B, Kyoto, Japan). Figure 1 shows the processing flow of nanocomposites. The ratio of CNF to PP used in this study was 1 to 5 wt.%, and MA-g-PP, which acts as a compatibilizer, was added at 3 wt.% based on the weight of PP. Both PP and MA-g-PP were firstly melted at 170 °C, followed by the addition of CNF suspension. A rotating speed of 50 rpm was used throughout the extrusion (Figure 1a). The PP/CNF nanocomposite produced was extruded out through die as strands and cooled prior to compression molding.

#### 2.4.2. Hot Compression

To form bar-shaped nanocomposites for mechanical testing, the strands were arranged in between steel molds (Figure 1b) and were placed on a hot compression molding machine (Type: Electrically heated platen press, Hsin-Chi Machinery Co. Ltd., Hsinchu Hsien, Taiwan) (Figure 1c). Nanocomposite samples were preheated in the mold at 170 °C for 1 min to allow complete melting at atmospheric pressure. A pressure of 110 kg/cm^2^ was then applied to the mold and held for 5 min under constant temperature to form nanocomposite sheets. The molded sheets were then transferred to a cold press and pressed for another 5 min. After that, they were transferred to a cold press and pressed for another 5 min. For a tensile test, 20 g of sample was used to form a nanocomposite sheet with a dimension size of 100 mm × 100 mm and 1 mm thickness. For a flexural test, 80 g of sample was used to form a nanocomposite sheet with a dimension size of 150 mm × 150 mm and 3 mm thickness (Figure 1d).

### 2.5. Characterization

#### 2.5.1. Morphological Analysis

Morphological analysis was conducted by two methods: visual appearance and scanning electron microscopy equipped with energy dispersive spectroscopy (SEM-EDS) (JCM 6000, JEOL Ltd., Tokyo, Japan). The fractured surfaces of the nanocomposite were observed by SEM-EDS to examine the distribution of oxygen element in the nanocomposite, which could represent CNF dispersion in the PP matrix. Prior to SEM-EDS analysis, samples were firstly coated with platinum using a vacuum sputter coater.

#### 2.5.2. Mechanical Performance 

Mechanical analysis consisting of tensile and flexural tests was performed using an Instron Universal Tester (Model 4302), Norwood, MA, USA. The tensile test was measured according to the ASTM Standard Method D638 on dumbbell shape specimens with 1 mm thickness at 5 mm/min crosshead speed and 30 kN of a load cell. The results were expressed in terms of tensile strength, tensile modulus, and elongation at break. Meanwhile, the flexural test was performed according to ASTM D790 on rectangular standard samples (dimension size of 120 mm × 12.7 mm and 3 mm thickness) and 30 kN of load cell. The support span length used was 56 mm and 1.45 mm/min crosshead speed. The flexural test was determined by a three-point bending test. The results were expressed in terms of flexural strength and flexural modulus. Both tensile and flexural tests were performed on five specimens for each formulation and the average values and standard deviations were reported.

#### 2.5.3. Crystallinity Analysis

The crystallinity analysis of the PP/CNF nanocomposite in this study was conducted using an X-ray powder diffractometer (Rigaku Corporation, Tokyo, Japan) equipped with a nickel Cu Kα radiation source (λ = 0.1542 nm) at 50 kV and 300 mA. The diffractograms were detected in the range 2*θ* = 5 to 50° at room temperature. The crystallinity index (CrI) was calculated based on Equation (1):

(1) $$CrI\;=\;\frac{\;I002-I\mathrm{am}}{I002}\;\times \;100\%$$

Note that *I*_002_ at an angle of 2*θ* = 23 and *I*_am_ at an angle of 2*θ* = 18 correspond to the cellulose and amorphous region, respectively [37].

#### 2.5.4. Thermal Properties Analysis

Thermal properties such as melting temperature *T_m_*, crystallization temperature *T_c_*, and Δ*H_m_* were determined using a differential scanning calorimetry (DSC). DSC measurements were performed using a Pyris 1 DSC calorimeter (Perkin–Elmer Co., Waltham, MA, USA). The samples were first heated from 30 to 200 °C at a rate of 10 °C/min and held at 200 °C for 1 min. They were then cooled to 50 °C at a rate of 10 °C/min and held at 50 °C for 1 min. Next, they were again heated to 200 °C at a rate of 10 °C/min and held at 200 °C for 1 min. The temperatures corresponding to the exothermic and endothermic peaks in the first heating step and the cooling step were called *T_m_* and *T_c_*, respectively. Δ*H_m_* values were determined from the areas of the melting and crystallization peaks, respectively. The Δ*H_m_* value was converted on the basis of the PP weight ratio of the nanocomposite.

#### 2.5.5. Thermal Stability Analysis

The thermogravimetric analysis was performed using a Thermogravimetry Analyzer (TGA) (TGA–9, Perkin Elmer, Waltham, MA, USA) under nitrogen flow. The sample (5–11 mg) was placed on a ceramic pan and set on the TGA. The sample was heated at a heating rate of 10 °C min^−1^ within the temperature range of 50–550 °C. From this analysis, the thermal stability and decomposition temperature of the PP/CNF nanocomposite were evaluated.

## 3. Results and Discussion

### 3.1. Morphological Analysis of PP/CNF Nanocomposite

**Figure 2:**
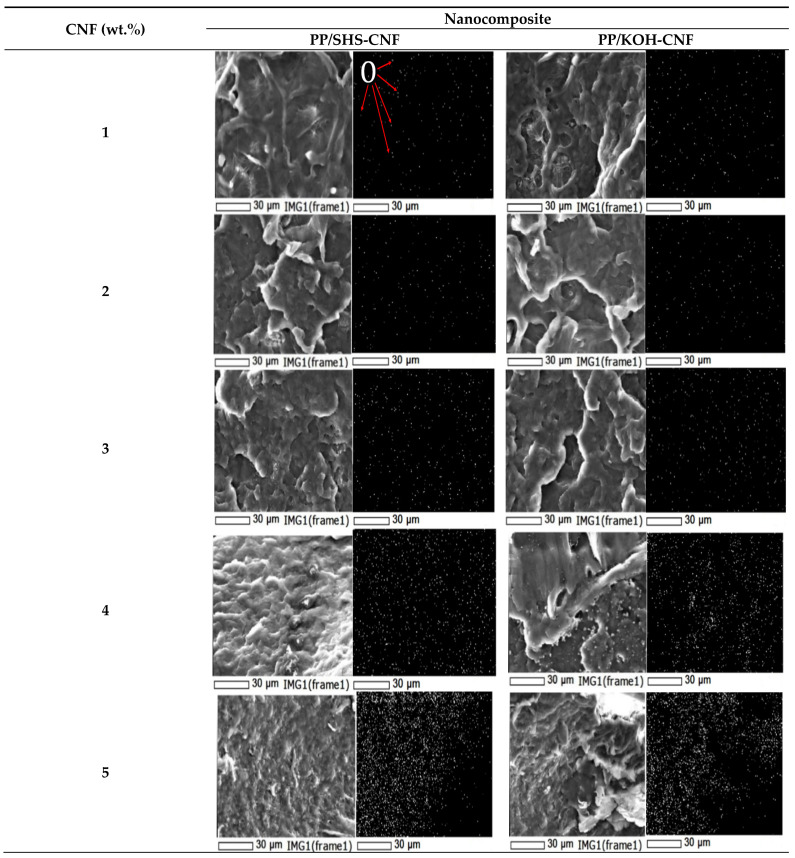
SEM-EDS micrographs of PP/SHS-CNF and PP/KOH-CNF nanocomposites. White dots in the EDS micrographs indicate the distribution of oxygen element being labelled by O in the figure.

In order to determine the effect of hemicellulose residue on the dispersion and distribution of CNF within the PP matrix, SEM-EDS analysis was conducted. Figure 2 shows the SEM-EDS images of the fractured samples of PP/SHS-CNF and PP/KOH-CNF nanocomposites, respectively. The EDS analysis was conducted to detect the distribution of oxygen element as a representative of CNF distribution, and it was represented by white spots in the EDS images [10]. From the SEM-EDS images, the PP/SHS-CNF nanocomposite exhibits better CNF dispersion and distribution up to 4 wt.% CNF, compared to the PP/KOH-CNF nanocomposite which only showed good dispersion and distribution up to 3 wt.% CNF. This observation could be explained by the presence of hemicellulose in SHS-CNF which prevented the CNF being agglomerated.

### 3.2. Mechanical Performance of PP/CNF Nanocomposite

**Figure 3:**
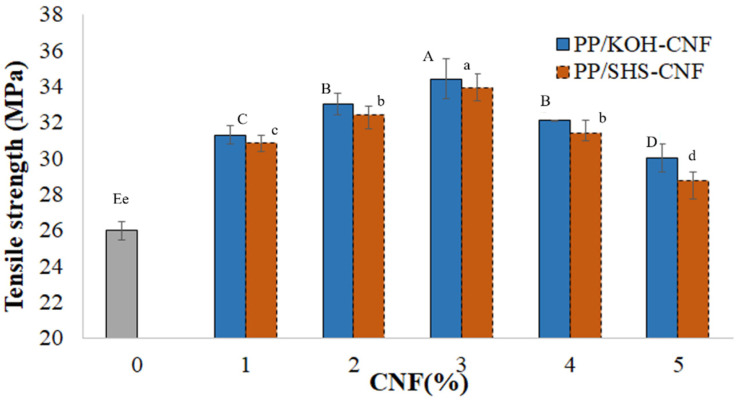

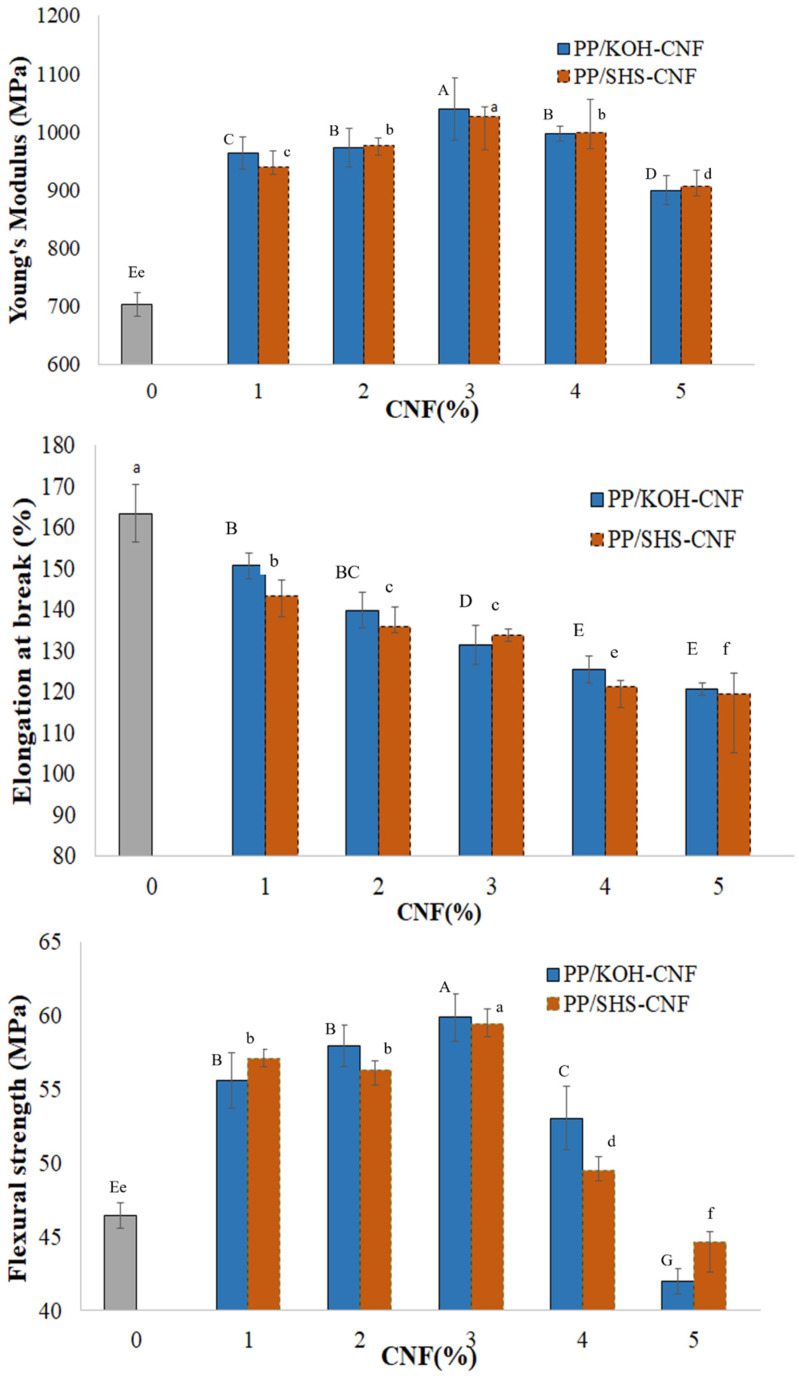

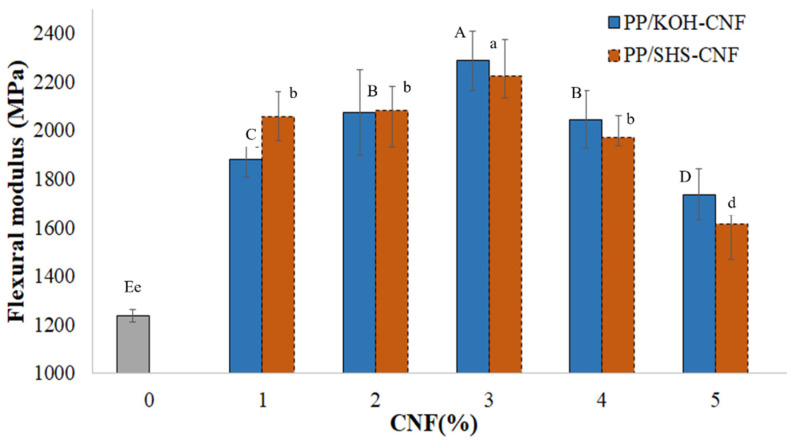
Mechanical performance of PP/CNF nanocomposite prepared from CNF suspension (1 to 5 wt.%). All data are means of 5 replicates ± S.D. Capital letters indicate significant difference (*p* < 0.05) among the PP/KOH-CNF nanocomposites, while small letters indicate significant difference (*p* < 0.05) among the PP/SHS-CNF nanocomposites according to one-way ANOVA and Duncan’s multiple range test.

The increment in mechanical properties was observed when CNF was incorporated in the PP matrix as shown in Figure 3 and Table 1. The results revealed that the tensile strength improved by 31–32% after 3 wt.% CNF was incorporated in the PP matrix for both SHS-CNF and KOH-CNF. This could be attributed to the homogenous distribution of CNF in the polymer matrix and the chemical composition of pretreated CNF [33]. Theoretically, the mechanical properties of a nanocomposite may increase by increasing the fiber loading in the polymer matrix [38,39,40]. Nevertheless, the mechanical performance of the nanocomposite was reduced after the incorporation of 4 and 5 wt.% CNF. This could be related to the dispersion of CNF in PP, as discussed before. The aggregated CNF in the nanocomposite was clearly seen at a high amount of CNF loaded in the matrix. From Figure 2, aggregation can be observed after 4 wt.% CNF was incorporated in the PP matrix. According to Yin et al. (2018) [41] and Feng et al. (2017) [42], the aggregation of CNF can act as stress-concentration sites, which causes poor stress transfer in the matrix and eventually reduces the mechanical performance of the nanocomposite [43,44]. 

Both SHS-CNF and KOH-CNF exhibited almost similar mechanical performance. The statistical analysis shown in Figure 3 revealed that there was no significant difference in the mechanical performance of PP/SHS-CNF and PP/KOH-CNF nanocomposites. For example, the tensile strength of PP/SHS-CNF (3 wt.%) and PP/KOH-CNF (3 wt.%) nanocomposites was 33.93 ± 0.8 MPa and 34.42 ± 1.1 MPa, respectively. Similarly, the flexural strength between these nanocomposites was found not significant as the PP/SHS-CNF (3 wt.%) nanocomposite had a flexural strength of 59.46 ± 1.0 MPa; meanwhile, the PP/KOH-CNF (3 wt.%) nanocomposite recorded an almost similar value at 59.88 ± 1.6 MPa. This indicates that the presence of 11 wt.% hemicellulose in SHS-CNF did not significantly influence the mechanical performance of the nanocomposite.

It was reported that the presence of hemicellulose and lignin could improve the mechanical performance of nanocomposites. This is because hemicellulose and lignin are believed to improve the chemical interaction of lignocellulosic fibers with the polymer matrix [45]. Nevertheless, it depends on the amount of hemicellulose/lignin in the fiber and the type of the polymer matrix used. A too high amount of hemicellulose/lignin could interrupt the interaction between fibers and the polymer matrix, thus reducing the mechanical performance [33].

### 3.3. Crystallinity of PP/CNF Nanocomposite

**Figure 4:**
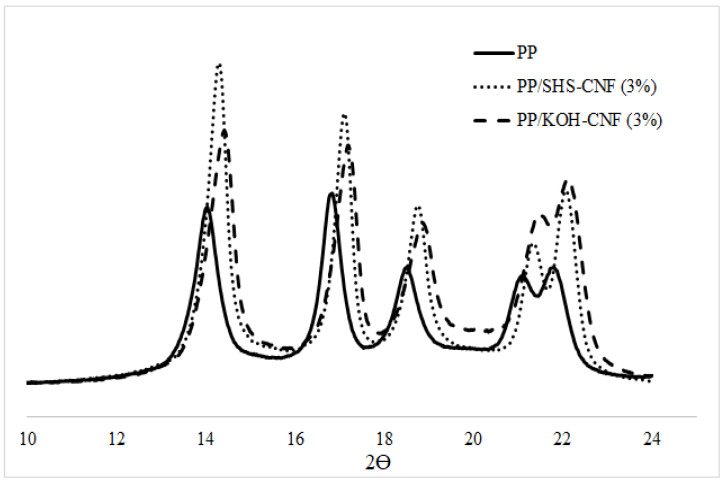
Crystallinity diffractogram of PP, PP/SHS-CNF (3 wt.%) and PP/KOH-CNF (3 wt.%) nanocomposites.

The XRD diffractograms of neat PP and PP/CNF nanocomposites are shown in Figure 4 and Table 2. PP was characterized by four main peaks at about 14°, 17°, 18°, and 22°. It can be seen that the addition of CNF causes a significant shift in Bragg’s angle. This shift and variation in interplanar distances might be attributed to the crystallite size variations of CNF. The incorporation of CNF into the PP matrix results in different long-range compressive forces on the crystals and unit cells, hence this explains the shifted diffraction peaks of PP/CNF nanocomposites [46,47]. All PP/CNF nanocomposites showed a higher crystallinity index (CrI) value than neat PP, which explains the better mechanical performance of the nanocomposite compared to neat PP. According to Shalwan and Yousif (2014) [48], Essabir et al. (2016) [49] and Sharip et al. (2021) [50], the CrI of the nanocomposite changed by self-nucleation effect (homogenous nucleation) and/or by a nucleating agent (heterogeneous nucleation). Hence, in this case, it is considered that CNF acted as a nucleating agent and produced seed crystals, resulting in the promotion of secondary crystallization and finally increasing the CrI value of the nanocomposite [51]. By comparing the CrI value of both nanocomposites, the crystallinity index of the PP/SHS-CNF nanocomposite was slightly lower than the PP/KOH-CNF nanocomposite. This could be explained by the presence of hemicellulose, which influenced the crystallinity by increasing the amorphous region of the CNF.

### 3.4. Thermal Properties of PP/CNF Nanocomposite

**Figure 5:**
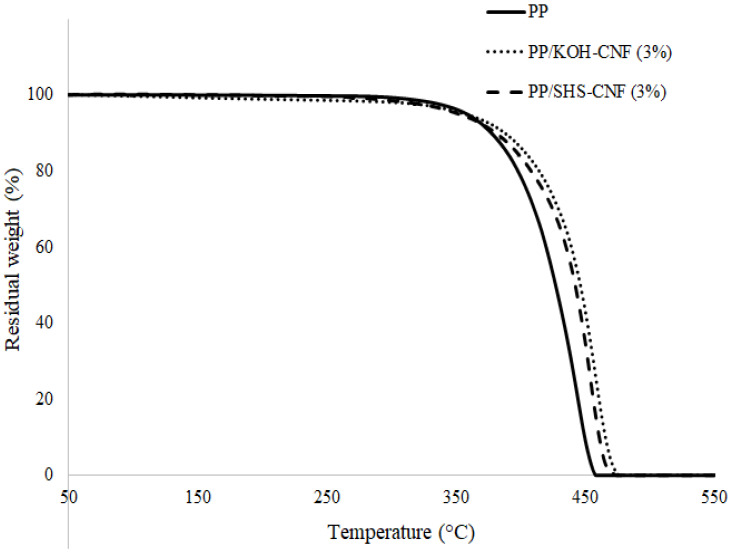
Thermograms of PP, PP/SHS-CNF (3 wt.%) and PP/KOH-CNF (3 wt.%) nanocomposites.

Thermal stability was evaluated based on the decomposition temperature at 5% weight loss (*T*_d5%_), 10% weight loss (*T*_d10%_) and 50% weight loss (*T*_d50%_) of the samples. Data for the thermogravimetry (TG) thermogram are shown in Figure 5 and Table 3. All thermograms exhibited no weight loss at temperatures lower than 200 °C, indicating that CNF is thermally stable up to 200 °C. The *T*_d5%_ and *T*_d10%_ values were recorded for each composite sample as they represent the initial degradation temperature derived from the reinforcing material in a polymer matrix [26]. The PP/SHS-CNF nanocomposite was slightly unstable at the beginning, where the *T*_d5%_ value was lower as compared to the PP and PP/CNF-KOH nanocomposite. This could be explained by the presence of hemicellulose in the SHS-CNF sample. Hemicellulose is known to be amorphous and less thermally stable than cellulose. It has a lower degradation temperature as compared to cellulose and PP.

Thermal properties were evaluated based on the *T_m_*, *T_c_* and Δ*H_m_* as listed in Table 4. The *T_m_* of all samples is similar. However, the *T_c_* value increased with the addition of CNF. This indicates that the presence of CNF affected the crystallization behavior of nanocomposites, in which the CNF acts as a nucleating agent in nanocomposites. The function of CNF as a nucleating agent has been reported elsewhere [26,50]. Both PP/SHS-CNF and PP/KOH-CNF nanocomposites showed a similar *T_c_* value, indicating that the presence of hemicellulose did not interfere with the function of CNF as a nucleating agent. Besides that, the hemicellulose content in SHS-CNF is not much as compared to KOH-CNF. It can be concluded that the size of the nanofiller is more important to influence the nucleating effect of the nanocomposite than its component.

### 3.5. Comparison of the Overall Performance of PP/SHS-CNF and PP/KOH-CNF Nanocomposite

The overall characteristics of PP/SHS-CNF and PP/KOH-CNF nanocomposites are compared in Table 5. Based on our previous study, the production of CNF from SHS-treated fiber was improved in terms of the total number of cycles, in which earlier nanofibrillation was observed as compared to KOH-CNF. This can be explained by the prevention of fiber agglomeration in the presence of hemicellulose. This reflects the improved productivity of CNF when SHS treatment was used. Additionally, the advantage of using SHS is its contribution towards the reduced use of chemicals. In terms of the performance of SHS-CNF, it is evidenced from this study that the performances of SHS-CNF are comparable to those of KOH-CNF (Figure 5). It is very interesting to note that the presence of residual hemicellulose did not interfere with the mechanical and crystallinity properties of the PP/CNF nanocomposite. A slight reduction was seen for thermal stability (*T*_d5%_), which is explained by the presence of thermally unstable hemicellulose.

## 4. Conclusions

The performance of CNF with residual hemicellulose as a reinforcement material is clarified in this study. There was no significant difference in the mechanical performance of PP/SHS-CNF and PP/KOH-CNF nanocomposites, indicating that the presence of some amount of amorphous hemicellulose in the CNF did not affect its function in improving the mechanical properties of the polymer. With the advantage of producing CNF at higher productivity and with less chemical use, the presence of hemicellulose in the CNF sample is proposed as the way forward in CNF application in nanocomposite application, towards producing more environmentally friendly nanocomposites without compromising the performance of the nanocomposite. 

## Figures and Tables

**Table 1:** Modification on the mechanical performance of nanocomposites as compared to neat PP.

Characteristics	Neat PP	PP/SHS-CNF (3 wt.%)	PP/KOH-CNF (3 wt.%)	Improvement (%)
Tensile strength (MPa)	25.99 ± 0.5	33.93 ± 0.8	34.42 ± 1.1	~31
Young’s Modulus (MPa)	703 ± 21	1027 ± 17	1040 ± 54	~46
Elongation at break (%)	163.43 ± 7.1	133.77 ± 1.6	131.33 ± 4.71	~−19
Flexural strength (MPa)	46.43 ± 0.8	59.46 ± 1.0	59.88 ± 1.6	~28
Flexural modulus (MPa)	1237 ± 27	2223 ± 150	2287 ± 122	~80

**Table 2:** Crystallinity index of PP, PP/SHS-CNF and PP/KOH-CNF nanocomposites.

Composition	Crystallinity Index (CrI) (%)
PP	50.1
PP/SHS-CNF (3 wt.%)	55.2
PP/KOH-CNF (3 wt.%)	56.0

**Table 3:** Thermal stability of PP/CNF nanocomposites.

Sample	*T*_d5%_ (°C)	*T*_d10%_ (°C)	*T*_d50%_ (°C)
Neat PP	356	377	427
PP/SHS-CNF (3 wt.%)	352	381	442
PP/KOH-CNF (3 wt.%)	358	387	446

**Table 4:** Thermal properties of PP/CNF nanocomposites.

Composition	*T_m_* (°C)	∆*H_m_* (mJ/mg)	*T_c_* (°C) (Onset)
Neat PP	162	99	122
PP/SHS-CNF (3 wt.%)	162	107	127
PP/KOH-CNF (3 wt.%)	162	106	126

**Table 5:** Comparison of properties of PP nanocomposites produced from SHS- and KOH-CNF.

Properties	PP/SHS-CNF	PP/KOH-CNF	Reference
Pretreatment of cellulose	Partially chemical	Totally chemical	[36]
Hemicellulose content	11.40 ± 1.9	0.00 ± 0.0	[12]
• Number of WDM cycles to produce CNF	6	8	[36]
Mechanical performance			
• Best CNF ratio (%) in nanocomposite	3	3	This study
• Tensile strength (MPa)	33.93 ± 0.8 ^a^	34.42 ± 1.1 ^a^	This study
• Flexural strength (MPa)	59.46 ± 1.0 ^a^	59.88 ± 1.6 ^a^	This study
• Young’s modulus (MPa)	1027 ± 17 ^a^	1040 ± 53 ^a^	This study
• Flexural modulus (MPa)	2223 ± 150 ^a^	2287 ± 122 ^a^	This study
Crystallinity index (%)	55.2	56.0	This study
Thermal stability at			
• *T*_d5%_ (°C)	352	358	This study
• *T*_d10%_ (°C)	381	387	This study
Function as nucleating agent	Yes	Yes	

^a^ All data of mechanical performance are means of 5 replicates ± S.D. A similar small alphabet superscript indicates no significant differences among the nanocomposite samples, at *p* < 0.05.

## Data Availability

The data presented in this study are available on request from the corresponding author.
